# Liquid Chromatography Mass Spectrometric Method and a Fluorometric 96 Well Plate Assay for Determination of Thiamine in Salmonid Eggs

**DOI:** 10.1021/acsomega.4c05862

**Published:** 2024-09-27

**Authors:** Manne Larsson, Lennart Balk, Elin Dahlgren, Efstathios Vryonidis, Dennis Lindqvist

**Affiliations:** † Department of Environmental Science, Stockholm University, SE-106 91 Stockholm, Sweden; ‡ Department of Aquatic Resources, Institute of Freshwater Research, Swedish University of Agriculture, SE-178 93 Drottningholm, Sweden

## Abstract

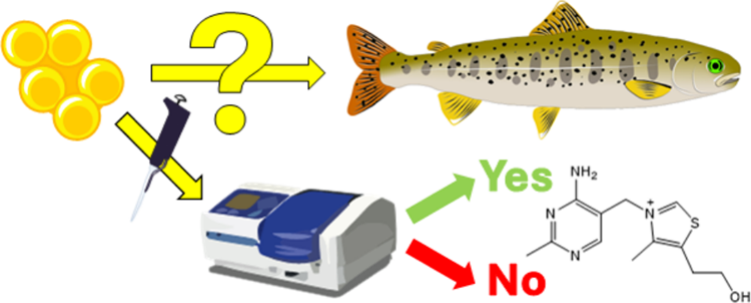

Thiamine deficiency is a large contributor to reduced reproduction success among salmonids throughout the northern hemisphere. In Scandinavia, this reproduction disorder is known as M74; while in North America, it is known as early mortality syndrome (EMS). The disorder fluctuates in magnitude from year to year. During years with high prevalence of the disorder, salmonid hatcheries that stock various aquatic systems to maintain the population size experience difficulties filling their quotas without thiamine treatment of alevins. The disorder is monitored both by observing the survival rate and by measuring the thiamine content of prefertilized eggs in the hatcheries. Here, a simple extraction procedure is presented, which allows for quantitative determination of the various phosphorylated forms of thiamine using liquid chromatography mass spectrometry but also allows for extraction in 96 deep-well plates and measurement of the total thiamine content using fluorescence monitoring with a plate reader, following oxidation of thiamine to thiochrome. The latter procedure could also be integrated into a highly portal system where the thiochrome is determined using the DeNovix QFX analyzer. The newly developed extraction procedure and cleanup method for fluorescence measurement represent the most versatile and simple methods to date for monitoring of thiamine in salmonid eggs. The methods produced accurate and precise data with quantification limits below the limit where the deficiency causes 100% lethality.

## Introduction

Thiamine, or vitamin B1, is indispensable to the health of all life.^1^ It plays a vital role as a cofactor in several life-sustaining enzymes and is required for cellular metabolism.^2^ Grave deficiency of thiamine in humans results in the sicknesses known as beriberi, Wernicke encephalopathy, and Korsakoff syndrome.^3^ Except for some fungi and bacteria, heterotrophs are dependent on thiamine intake via food.^1,4^

In the 1970s, at the time, unexplained death of salmon (*Salmo salar*) fry was observed in hatcheries in Sweden. Unprecedented numbers of the yolk sac fry did not survive to become smolt. This was labeled the M74-syndrome, and has been recurring ever since, to a varying degree.^5,6^ It was not until the 1990s when similar phenomena in North America, “early mortality syndrome” (EMS) in lake trout (*Salvelinus namaycush)*, native to the Great Lakes,^7^ and “Cayuga syndrome” in landlocked atlantic salmon (*S. salar*) of the Finger Lakes,^8^ was investigated, the connection to thiamine deficiency was made. It was also during this decade that the highest mortality of M74 was observed in the Baltic salmon stocks with up to 90% offspring mortality in certain rivers.^5^

The impact of thiamine deficiency on the Baltic salmon population is still a concern, and hence it is monitored annually in Sweden and Finland.^6^ Unfortunately, thiamine deficiency is not a localized phenomenon and is not limited to salmonids. Apart from the Baltic Sea, and the previously mentioned American lakes,^7,8^ thiamine deficiency in salmonids has been reported in Alaska,^9^ and recently in California.^10^ Thiamine deficiency has also been reported in other fish, such as cod^11^ and eel, as well as in mussels, birds,^12^ and reptiles.^13^

Most chemical methods for thiamine detection and quantification rely on the possibility to derivatize thiamine (and its phosphate esters) to the fluorescent thiochrome.^14^ Chromatographic separation of the congeners is achieved on reversed phase columns, most often using phosphate buffers in mobile phases.^15^ Recently, a few liquid chromatography-mass spectrometry (LC-MS)-based methods have been developed, achieving chromatographic separation using ion-pairing agents (IPAs) compatible with electrospray ionization (ESI).^16,17^

Previous extraction methods for thiamine include steps of boiling and/or the use of strong acids, for instance hydrochloric acid, sulfuric acid, or trichloroacetic acid (TCA), which has been the norm for accurate determination of thiamine deficiency over the past decades.^14^ These steps are time-consuming and risk the formation of oxythiamine.^18^ A rapid fluorescence (FL) method for determination of thiamine in salmon roe was developed by Zajicek et al. making use of solid-phase extraction to rid the matrix of constituents interfering with fluorescence detection.^19^

To fully grasp the magnitude of the problem with thiamine deficiency in the aquatic system and monitor its development, there is a need for simple, cheap, reliable, and fast analytical methods. The aim of this paper was hence to develop methods for analysis in roe to cover all situations: precise determination of the levels of thiamine and its phosphorylated esters, analyses of large numbers of samples, and analyses of samples in the field or within salmon hatcheries.

## Materials and Methods

### Samples

All samples used in this study came from aliquots of roe collected in Atlantic salmon hatcheries for the monitoring of thiamine. No samples were specifically collected for this study, and hence no additional ethic approval was required. The samples used here came from three different Swedish rivers: Dalälven, Umeälven, and Skellefteälven (Kvistforsen), and were collected over the years 2020–2022.

### Chemicals and Reagents

All solvents were of analytical grade quality and were purchased from established brands. Thiamine hydrochloride (Thia), thiamine monophosphate chloride dihydrate (TMP), thiamine diphosphate (TDP), amprolium hydrochloride, and thiochrome were all of analytical and pharmaceutical standard grade and were purchased from Sigma-Aldrich (Saint Louis, MO) (certified reference material). Stock solutions of standards were prepared from crystals in concentrations of 2–3 mmol/L in hydrochloric acid (HCl; 0.1 M) and kept dark at 4–8 °C. Working solutions, in HPLC-grade water, were prepared weekly and kept dark at 4–8 °C, in polypropylene (PP)-tubes. *N*,*N*-Diisopropylethylamine (Hünig’s base; >99.5%) was from Acros Organics (Geel, Antwerpen, Belgium). Potassium hexacyanoferrate(III) (K_3_[Fe(CN)_6_]) of analytical grade was purchased from Merck (Darmstadt, Germany). Saturated dipotassium hydrogen phosphate (K_2_HPO_4_) was made by dissolving water-free K_2_HPO_4_ (14 g) from VWR (Radnor, PA) in liquid chromatography grade water (10 mL), in two steps (7 g + 7 g).

### LC-MS-Based Method

#### Extraction

Frozen salmon roe (1 g, ∼6–11 eggs) was weighed in a 10 mL PP test tube. Surrogate standard solution (amprolium 60 μL, 100 μM) and cold acetone (−20 °C, 2 mL) were added, after which the sample was homogenized swiftly (<10 s), using a bench mixer (IKA T25 basic Ultra-Turrax) at 20,000 rpm. Water (of HPLC-grade, 4 mL) was then added, and the mixture was homogenized again (5 s), before addition of dichloromethane (DCM; 4 mL). The test tube was shaken and centrifuged at 3000 rpm for 3 min. The aqueous upper phase was transferred to a new 10 mL PP test tube. The pellet and organic phase were re-extracted using water (2 mL), by shaking and centrifugation, as above. Note that DCM can have a deteriorating effect on PP test tubes and hence the sample should not be left with DCM for prolonged periods. The water fractions were pooled together, and the test tube containing DCM and pellet was discarded. The pooled water extracts were washed twice with DCM (4 mL + 3 mL). An aliquot of the water extract was filtered through a syringe filter (0.20 μm), and 0.5 mL was collected in a plastic LC vial. The vial was then placed under stream of nitrogen gas, inside a fume hood, for 15–20 min to remove residual organic solvent, and the sample was subsequently diluted with water to 1.5 mL before instrumental analysis.

#### Instrumental Analysis

All analyses were performed on an ACQUITY UPLC system, coupled to a Xevo TQ-S micromass spectrometer (Waters Corporation, Millford, MA). Waters Masslynx software (version 4.1) was used to control the systems and process data. An XBridge (Waters) Phenyl column (2.1 × 100 mm^2^, 3.5 μm) at 30 °C was used to achieve chromatographic separation of the analytes. The autosampler was set to 15 °C and the injection volume was 2 μL. Mobile phases used were (A) 10 mM Hünig’s base in 5% MeOH, adjusted to pH ∼ 8.2 with acetic acid, and (B) MeOH. The flow rate was 0.4 mL/min, and the gradient conditions were 0.00–1.20 min 0% B, 1.70–2.90 min 20% B, 4.50–5.80 min 45% B, 6.50–10.00 min 0% B. Ionization was performed by electrospray in positive mode (ESI^+^) and detection by multiple reaction monitoring (MRM). The capillary voltage was set to 2.80 kV, the desolvation temperature was set to 600 °C, the desolvation flow was set to 700 L/h, and the cone flow was set to 40 L/h. MRM transitions as well as cone voltages and collision voltages are presented in the Supporting Information (SI; Table S1 and Figure S1).

#### Recovery

Recovery was determined in three different levels, low (Thia: 20 μM, TMP: 2 μM, and TDP: 4 μM), medium (Thia: 140 μM, TMP: 8 μM, and TDP: 16 μM), and high (Thia: 360 μM, TMP: 20 μM, and TDP: 40 μM), by spiking 50 μL of the respective test solution into samples with low native levels of thiamines. The recovery standard was spiked prior to extraction, and the volumetric standard, amprolium, was added after the extraction prior to aliquotation for instrumental analysis. To correct for the native content of thiamines, eggs from the same sample were also extracted for the reference, where the test solution spikes were added together with the volumetric standard prior to instrumental analysis. The spiking levels were selected to be representative of natural levels, where, for instance, the low level, mentioned above, corresponds to concentrations typical for roe very low in thiamine (Thia: 1 nmol/g, TMP: 0.1 nmol/g, and TDP: 0.2 nmol/g), and the high corresponds to a replete sample (Thia: 18 nmol/g, TMP: 1.0 nmol/g, and TDP: 2.0 nmol/g). Note that this high level is exaggerated for TMP and TDP, as their concentrations in samples do not vary as much as thiamine does (see Results and Discussion). Fifteen individual extractions were made for each level, with an additional six extractions being made, where the re-extraction step of the eggs was omitted.

#### Accuracy and Precision

Accuracy was determined both by comparing the results of the sum of thiamines (SumT) with that given by the fluorescence-based method and by intercalibration against another laboratory (Finnish Food Authority) for the determination of thiamine in 20 samples. The Finnish Food Authority uses the method described by Vuorinen et al.,^20^ which in turn is based on the well-established method by Brown et al.^21^ Statistically significant bias between methods were tested for in excel, using two-sided paired *t*-test, where each individual sample was paired between the methods. The *t* tests were proceeded by a Kolmogorov–Smirnov test for normality among the differentials between the methods.

Precision was evaluated by conducting eight analyses each of roe samples from three different females and calculating the coefficient of variance (CV%) for each of thiamine, TMP, and TDP.

#### Detection Limits and Linearity

Limit of detection (LOD) and limit of quantification (LOQ) were calculated from the lower part (eight concentrations) of the calibration curve according to the International Conference of Harmonization (ICH) guideline, with LOD calculated as 3.3, and LOQ 10, times the standard error of estimates (using the STEYX formula in excel) divided by the slope (Figure S2). Method limits of detection and quantification (MLOD and MLOQ) were calculated considering a 3× dilution of the total volume of added water (6 mL) and adjusting for the average determined recoveries for the different forms, respectively.

### Fluorometric 96 Well Plate Assay

#### Extraction and Oxidation to Thiochrome

Two eggs were placed in each well of the deep-well plate (96/2000 μL, square wells, Eppendorf, Hamburg, Germany). Acetone (300 μL) was added before mechanically crushing the eggs with an 8-channel zinc plated plunger (see Figure S3 in the SI). Water (600 μL) followed by DCM (600 μL) was then added, and the sample solution was mixed by gently plunging up and down. The plate was centrifuged at 2000*g* for 3 min, before transferring 200 μL of the top aqueous phase to a new deep-well plate. The thiamine was oxidized to thiochrome (TiOC) by the addition of K_3_[Fe(CN)_6_] (0.35%, 50 μL) in potassium hydroxide (0.5 M). The reaction was quenched after a few seconds by the addition of saturated K_2_HPO_4_ (400 μL) followed by isopropanol (IPR; 600 μL). The solution was mixed by aspiration with an 8-channel pipet, making the analytes shift phase, from the saturated water, into the IPR. The plate was centrifuged as above, before transferring 200 μL of the top IPR phase to a normal 96 well plate (Costar black plate, clear bottom, Corning Inc., Kennebunk, ME) for fluorometric analyses.

During quantitative analyses, the first column of the deep-well-plate was assigned for background and calibration. A stock solution of thiamine was prepared in 0.1 M hydrochloric acid in a PP test tube before diluting to a working stock solution of 20 μM in water. The working stock solution was further diluted in seven steps to 17, 12, 8, 5, 3, 2, and 1 μM, 100 μL of each calibration level was added in a rising order in the first column of the 96-well plate, and 100 μL of water was added to the very first well to serve as the blank. The calibration column was treated the same way as for the samples.

#### Instrumental Analysis

Fluorescence was measured using a SpectraMax iD3 plate reader (Molecular Devices, San Jose, CA), with excitation at 373 nm and emission at 439 nm. Fluorescence was read from the top at a height of 1 mm from the plate, and the integration time was 400 ms. Excitation and emission spectra as well as background absorbance spectra for both TiOC and K_3_[Fe(CN)_6_], used to optimize the wavelength settings, can be found in the SI (Figure S4).

#### Recovery and Optimization

The recovery of TiOC from samples relative to that of spiked procedural blanks (as used for calibration) was determined in 32 samples with different native concentrations of SumT. Each sample was analyzed five times, three times spiked with thiamine (2 nmol), and two times unspiked. The recovery was calculated by subtracting the FL signal of the unspiked sample from that of the spiked sample and dividing that with the FL signal of the background subtracted reference, i.e., spiked procedural blank (2 nmol thiamine), according to eq 1.
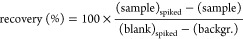
1The relative recovery of TMP and TDP compared to thiamine was determined by comparing the FL signal recorded from spiked procedural blanks, 8 per substance. The recovery of TiOC in the IPR partitioning step was evaluated at eight different concentrations.

The absolute recovery of the method is almost solely determined by the yield during oxidation and formation of TiOC. To determine the yield of the reaction and the influence that the matrix and the amount of reagent have on the yield, the formation of TiOC from thiamine was measured by comparing the FL signal with that of a native TiOC standard at equal molar concentration. The yield was determined both in blanks and in samples with different amounts of the reagent (K_3_[Fe(CN)_6_]). The absorbance spectra (330 and 455 nm) of the samples were also registered.

#### Accuracy and Precision

Accuracy of the 96-WP method was determined by comparing the quantitative results of SumT from 122 samples, collected from 3 different rivers and at 2 different years, with the corresponding results from the LC-MS method.

Precision was determined by conducting eight analyses of eight different samples and calculating the CV% for each sample. One of the samples was also analyzed individually 8 times using 2 mL μ-tubes instead of the deep-well plate.

#### Clinical Sensitivity and Specificity

By setting a reference value for thiamine deficiency and applying the LC-MS results as the true values, the clinical sensitivity and specificity were calculated according to eqs 2 and 3 respectively.
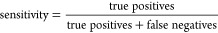
2
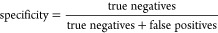
3

#### Detection Limit and Linearity

MLOD was calculated as 3×, and MLOQ 5×, the standard deviation (SD) of the background, was added to the average background signal. The background was based on 12 procedural blank analyses spread out over time. Linearity was assessed by analyzing procedural blanks spiked with thiamine at 13 different levels from 0.01 to 2 nmol, each concentration was run in triplicates.

Background and quenching of the signal were investigated by spiking TiOC (10 μM, 40 μL) to the final 200 μL from the extraction of 0, 1, 2, and 3 eggs (4 analyses per amount of eggs) and measuring the fluorescence signal as well as the absorbance at 373 and 439 nm.

### Portable Fluorometric Detection Option

As a portable option for the determination of thiamine, a DeNovix QFX fluorometer (DeNovix Inc., Wilmington, DE) was evaluated. The QFX has a UV excitation channel at 375 nm with an emission range of 435–485 nm. The QFX can only analyze one sample at the time and uses 0.5 mL μ-tubes (PCR tubes). Two different μ-tubes were evaluated: Standard graded 0.5 mL Eppendorf tubes (Eppendorf) and Axygen 0.5 mL thin wall, clear PCR tubes (Corning Inc.). Sensitivity and linearity were evaluated similarly to those of the iD3 plate reader. Sample preparation was done as with the 96-WP method, although with individual samples in 2 mL μ-tubes, and the final volume was also 200 μL. Accuracy was determined by comparing the quantitative results of SumT from 24 samples, collected from 3 different rivers (2022), with the corresponding results from the LC-MS method.

## Results and Discussion

### LC-MS-Based Method

#### Extraction Procedure

Thiamine is generally extracted from roe and other tissues using water, which requires strong acids to release thiamine and precipitate proteins and enzymes, such as in the long-established method described by Brown and co-workers,^21^ as well as the methods described by Körner^22^ et al. and Batifoulier^23^ et al. The extraction method presented here, on the other hand, was developed based on simple classical lipid extraction methods including the Folch,^24^ Bligh and Dyer,^25^ and Jensen methods.^26^ Denaturation was achieved by homogenization in acetone, as used in the Jensen method.^26^ Water and dichloromethane were then added, similarly to the Bligh and Dyer method, which utilized chloroform,^25^ causing the lipids and pigments, of which the roe is rich in, to distribute to the DCM phase leaving the thiamine in a clean water phase. The choice of using a chlorinated solvent was made to achieve an upper aqueous layer; this becomes essential particularly when progressing to a 96-WP method. It should be noted that DCM could be harmful to humans, and in the US stricter rules for its use have recently been enforced (United States Environmental Protection Agency Methylene Chloride; Section 6(a), Toxic Substances Control Act). Users of this method should follow safety regulations. Acetone was selected over alcohols due to its large distribution to the DCM. Solvent remnants in the water phase have a large effect on the subsequent LC-MS analysis; hence, all samples were placed under a stream of nitrogen to evaporate potential solvent remnants before instrumental analysis. Basing the extraction on lipid extraction methods, developed to quantitatively extract all lipids from the samples, resulted in an aqueous phase that was clean enough to even conduct analyses using spectrometric methods without the need for chromatography.

#### Instrumental Analysis

Determination of small, polar organic compounds such as thiamine and, particularly, its phosphate esters, using LC-MS, presents challenges due to low retention of the analytes in reversed phase systems. LC-MS is also not compatible with e.g., phosphate buffers, which is used in many published high-performance liquid chromatography (HPLC)-fluorescence methods for thiamine.^14,15,21^

By adding ion-pairing agents (IPA) such as aminoacetates to the mobile phase, retention and separation of the analytes can be achieved on a reversed phase column. Previously used IPAs for thiamine analysis include ammonium carbonate and ammonium/hexylammonium formate.^15,17^ Here, three IPA candidates were tested as their acetate salts, at different pH: *N*,*N*-diisopropylethylamine, (Hünig’s base), triethylamine, and ammonia. We chose to continue with Hünig’s base as it offered good separation, peak shape, and signal at a wide pH range.

Both TMP, and, particularly, TDP are amphoteric, with p*K*_a_ values that lie rather close.^27^ Thus, in order to elute each analyte as one single peak, the pH of the mobile phase needs to be high to minimize the occurrence of several acid/base-species for each analyte during chromatographic separation. To avoid damage to the column, it is important to choose columns that tolerate high pH values. The XBridge phenyl (Waters) column used here can sustain pH values up to 12.

There is a marked loss of signal when using vials and test tubes made of glass at pH higher than 2. A series of tests were made with standard solutions, comparing glass and plastic vials at different pH. Particularly thiamine and the internal standard (Amprolium) were observed to be very sensitive to glass, most prominently at pH 3–4. For instance, at pH 4, the loss of thiamine in a glass vial could be upwards of 50% within an hour and more than 90% after 4 h. The signal loss, likely caused by adsorption to the glass, is, at least partly, reversible by lowering of the pH. These results are in agreement with a recently published study investigating the effect of glass containers on thiamine.^28^ The extracted roe samples here have a pH of 6–7, and would, if run in glass vials, be highly affected by this effect. Thus, only plastic (PP) tubes and vials can be used with this method.

#### Recoveries

**Figure 1:**
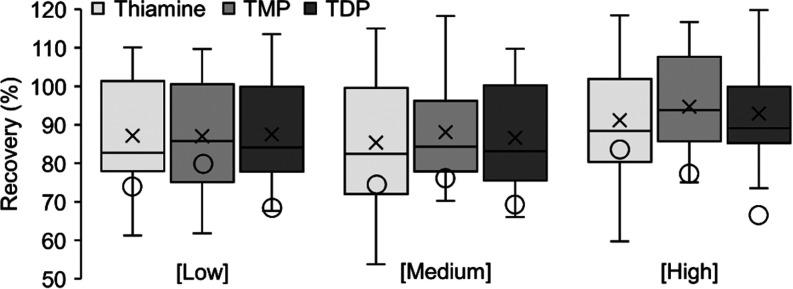
Recovery of thiamine, TMP, and TDP, respectively, at three different concentrations. The box and whiskers plot depict the recovery when including the re-extraction step. The circle depicts the average recovery when omitting the re-extraction step.

The recoveries were overall high with small variations. There was no statistically significant difference (two-sided, *t*-test) between the low and high concentrations for either thiamine (*p* = 0.54), TMP (*p* = 0.14), nor TDP (*p* = 0.27). Similarly, when grouping all test concentration together, no differences could be seen between the average recovery of thiamine (88 ± 18%) and the recovery of TMP (90 ± 14%; *p* = 0.55), respectively TDP (89 ± 14%; *p* = 0.74) (Figure 1). Omitting the re-extraction of the eggs, with the additional 2 mL of water, significantly reduced the recoveries and introduced a difference between the different forms of thiamine, with TDP dropping an additional 10% compared to thiamine and TMP (Figure 1). However, as TDP is less important when it comes to salmonid eggs, as thiamine constitutes the majority of the SumT, and the internal standard compensates for the extra loss when not conducting the re-extraction, this step can still be omitted to save time.

#### Accuracy and Precision

**Figure 2:**
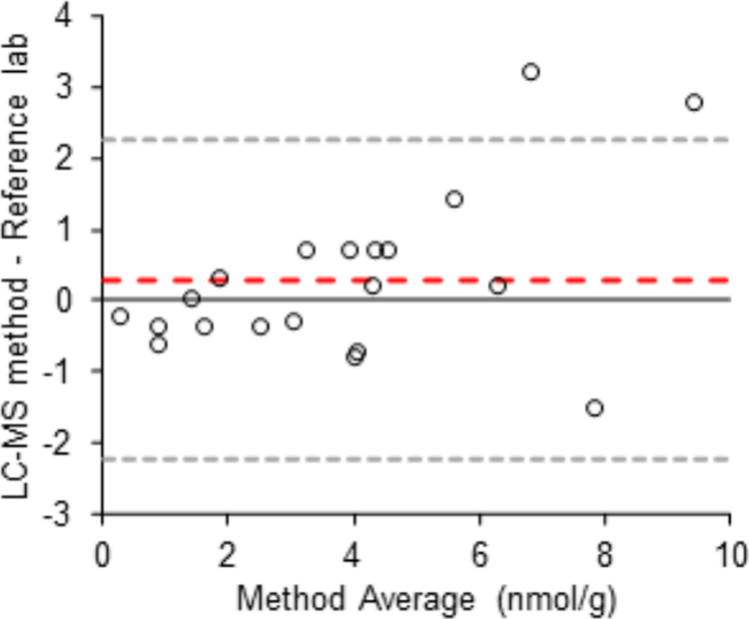
Bland–Altman plot depicting the differentials between the two methods plotted against the average of the methods. Red dotted line depicts the bias (average of the differentials), gray dotted lines depict the upper and lower limits of agreement (1.96× SD of the differentials).

Evaluation of the intercalibration using a Bland–Altman plot revealed a slight positive bias (Figure 2), and two samples were above the upper limit of agreement (calculated as 1.96× the standard deviation (SD of the differentials)). However, the bias was not statistically significant (*p* = 0.29, two-sided paired *t*-test), and the results deviated with more than ±25% from the average between the laboratories, in only 2 out of 20 samples (Table S2).

The precision was high with values for coefficient of variation (CV%) averaging 8, 7, and 12%, for thiamine, TMP, and TDP respectively, based on roe samples from three females each analyzed eight times (Table S3).

#### Detection Limits and Linearity

The LOD and LOQ calculated using the ICH formula did seem a bit high in relation to naturally occurring levels (Table 1). In fact, calculating the MLOQ on the signal-to-noise ratio (10× S/N), on a sample-to-sample basis, generally allows for determination of levels much lower than the MLOQ presented in Table 1. Of course, the LOD and LOQ determined by the ICH formula can be significantly improved by repeated measurement of the calibration standards and deriving average values, or by simply reducing the concentration span tested. Similar for all three forms, the dynamic range spanned 3 orders of magnitude. Above 2 μM and below 1 nM significant curvature was starting to show (Figure S2).

**Table 1:** Limit of Detection and Quantification (LOD, LOQ) for the Three Forms of Thiamine on the LC-MS System as well as Method LOD and LOQ for the Method Considering the Respective Recovery and Expressed as nmol/g

	thiamine	TMP	TDP	
LOD	1.8	1.8	2.9	nM
LOQ	5.6	5.4	8.6	nM
recovery	88%	90%	89%	
MLOD	0.04	0.04	0.06	nmol/g
MLOQ	0.11	0.11	0.17	nmol/g

### Fluorometric 96 Well Plate Assay

#### Recovery and Optimization

**Figure 3:**
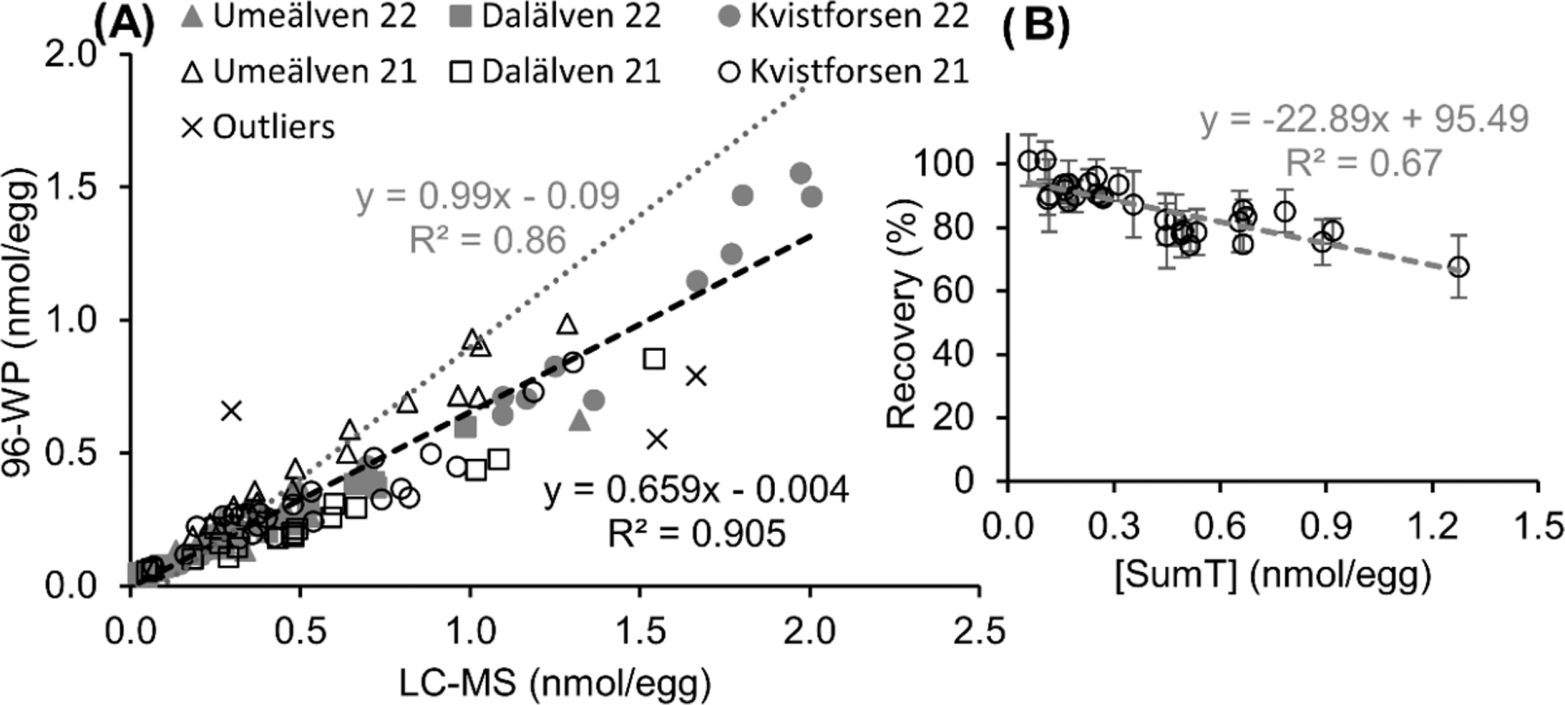
(A) Quantitative determination of SumT (nmol/egg) in 122 samples from 3 different rivers and 2 years, using the 96-WP method vs the LC-MS-based methods. The individual data points and black trendline are without recovery correction of the 96-WP method, while the gray trend line displays the correlation following recovery correction of the data set, according to the equation in (B). (B) Recovery of TiOC from thiamine-spiked samples (32 in total), with different levels of native SumT, relative to that of spiked procedural blanks (spiked water subjected to the same extraction and workup procedure as the samples).

The relative recovery of TiOC from thiamine-spiked samples in relation to that of thiamine-spiked procedural blanks was high (average 86%), but there was a clear decreasing trend in recovery with increasing native thiamine content of the eggs (Figure 3B). However, the recovery is not dependent on the actual thiamine content, but rather it is likely affected by covarying factors. For example, as increased thiamine content generally means increased size of the eggs (see Figure S5) and increased pigmentation,^29^ it is also likely to be correlated to the levels of other nutrients. Most likely, these factors decrease the yield of the oxidation, leading to the apparent observation that the increased thiamine content reduces the recovery. For example vitamin C is known to interfere with the oxidation by consuming K_3_[Fe(CN)_6_].^14^ This effect can be corrected for, by using a sliding recovery to correct the determined levels according to eq 4, based on the linear equation for recovery versus concentration in Figure 3B.
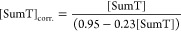
4The relative recovery between TMP and thiamine was 93%, indicating a negligible decrease in the recovery of TMP. However, the relative recovery of TDP was only 67%, indicating a clear and significant reduction in recovery relative to that of thiamine (see Table S4A). A reasonable source of the reduced recovery is the IPR partitioning step, where the two phosphate groups significantly increase the water solubility and decrease the solubility in the organic solvent. Note that this difference was not seen in the LC-MS based method, where the oxidation and IPR partitioning steps are not included. The recovery of TiOC in the IPR partitioning step was however quite high at 91% (see Table S4B). The partition of the TiOCs into IPR has several beneficial outcomes; besides reducing the background by removing the oxidation reagent residues, it also increases the stability of the samples, and storage even at room temperature for up to 1 month (in the dark) does not cause significant degradation of the samples (see Table S5).

The lower recovery of TMP and specifically TDP has little effect on the monitoring of the thiamine content in salmonid eggs. Not only do they make up only a small portion of the SumT in *S. salar* roe but they also fluctuate much less, showing fairly constant levels in the roe and poor correlation to the levels of thiamine (see Figure S6). This in accordance with the work by Vuorinen et al.^30^ It is probable that the nonphosphorylated form is kept as a reservoir in the roe, for embryonic development, and that the production of viable eggs always includes transfer of a certain amount of the active TDP form, hence leaving these levels fairly constant even in severe cases of thiamine deficiency.

The single most influential step of the method, with regards to the absolute recovery, is the oxidation step and formation of TiOC. The use of K_3_[Fe(CN)_6_] for this purpose is well established although other reagents are sometimes used.^14^ Due to competitive reactions, particularly the formation of thiamine disulfide, the oxidation to TiOC never reaches 100%.^14^ Here, it was clearly noticed that the yield increased with lower amounts of the reagent, and it was possible to back the concentration down to reach yields of over 60% in blank samples (Figure S7A). However, in samples one needs to increase the amount of reagent as the matrix consumes a large amount, which could be seen both in the amount needed to get the highest possible (and stable) fluorescence signal from the samples (Figure S7A), and in the reduction of the background absorbance spectra (Figure S7B). It was determined that the lowest amount that could be used in the reagent solution was 0.2% K_3_[Fe(CN)_6_], but as this varies to some extent between samples, 0.35% was used as a precaution. This in turn decreases the yield of the reaction in spiked procedural blank samples to around 40% (Figure S7A).

#### Accuracy and Precision

The SumT results provided by the 96-WP method from the 122 samples correlated well with those recorded using LC-MS (*R*^2^ = 0.905, see Figure 3A). However, even with the sliding recovery correction (gray dotted line and equation of Figure 3A), the 96-WP results deviated significantly from the LC-MS results when applying a paired *t*-test over the whole data set (*p* < 0.001). More often than not, the 96-WP method seemed to slightly underestimate the levels compared to the LC-MS method, but the average variation between the methods (23 ± 15%) was small in the context of monitoring thiamine content in salmonid eggs. It is of course possible to use the linear equation derived from the uncorrected 96-WP results vs LS-MS (black equation in Figure 3A), to tune the 96-WP results instead of using the sliding recovery, in which case, the results do not significantly differ between the methods (*p* = 0.86). However, the good correlation and small average variation in the results between the methods when using the sliding, or even the average, recovery correction indicate that the method is suitable to be introduced in monitoring as it is based on accuracy.

The precision (CV%) of the 96-WP method spanned from 11 to 23% with an average of 17% over the eight samples (each analyzed eight times). When conducting individual analyses in μ-tubes, the precision increased compared to the 96-WP for the same sample, from 19 to 7.8%, indicating a slightly better reproducibility. However, there was no significant difference in the average determined concentrations (*p* = 0.75, two-sided *t*-test) (see Table S6). Since the eight samples did not come from a homogenate but rather represent eggs (two for each sample) from the same female, the variation used to determine the precision also include the natural between egg variation in thiamine content. Hence, the low CV% values also indicate that the use of only two eggs is still representative of the average thiamine content of all of the eggs from the same female.

#### Detection Limit and Linearity

The thiamine concentration range of interest, based on the amounts found in *S. salar* eggs, was well within the dynamic range of the instrument, which produced more than adequate linearity (Figure S8). The detectability also proved adequate for the application with a calculated MLOD and MLOQ of 0.030 and 0.049 nmol, respectively (Figure S9), calculated based on nmol thiamine added to each well. As two eggs are used in the analysis, these figures would correspond to 0.015 nmol/egg (MLOD) and 0.024 nmol/egg (MLOQ). At levels close to MLOQ, the yield of TiOC from samples relative to that of thiamine-spiked blanks is close to 100% (Figure 3B), and since the suppression of the FL signal when using two eggs is negligible (Figure S10B), MLOQ values based on spiked blanks are likely quite equal to those of real samples.

Using the data published by Werner and co-workers on thiamine levels in unfertilized eggs and the subsequent embryo mortality in *S. salar* from the St. Marys river in MI,^31^ a lethal concentration 50% (LC_50_) of 1.12 nmol/g ww was calculated (Figure S11). Furthermore, there is a strong linear relationship between SumT expressed as nanomoles per gram and SumT expressed as nanomoles per egg (*R*^2^ = 0.95) in unfertilized *S. salar* eggs from the Baltic Sea, which allows for conversion between the units of measure (Figure S12). By using the linear equation in Figure S12, the LC_50_ can thus be recalculated to 0.10 nmol/egg, which is 4 times above the determined MLOQ. In fact, at or below the MLOQ level (0.024 or 0.58 nmol/g), the mortality rate is 100%, hence indicating little need for higher sensitivity.

Furthermore, in the data set published by Werner and co-workers,^31^ a background lethality of about 7% can be estimated, and a significant increase in mortality is observed at thiamine levels below 1.7 nmol/g or 0.19 nmol/egg (Figure S11). By using this value (0.19 nmol/egg) as a pathological limit for thiamine content in the eggs and by using the LC-MS values as the true levels, the clinical sensitivity (eq 2) and specificity (eq 3) for the 96-WP method could be calculated. With 36 true positives and 1 false negative, the clinical sensitivity was high, at 97%, while the specificity was calculated to 87%, with 82 true negatives and 12 false positives.

### Portable Fluorometric Detection Option

The DeNovix QFX fluorometer is a cheap and highly portable alternative to the plate reader, and although the rate at which samples can be analyzed is significantly reduced when conducting analyses in μ-tubes compared to deep-well plates, the precision did as mentioned improve. Furthermore, the instrument precision was high with little noise and good dynamic range, resulting in an improved MLOD and MLOQ at 0.006 and 0.009 nmol/egg, with the thin-walled, clear PCR tubes (calculated based on 14 procedural blank analyzes and an 8-point calibration curve with each level run in quadruplicates). The instrument could even be successfully used with standard 0.5 mL graded Eppendorf tubes, although the background became much higher, and the sensitivity (slope of the calibration curve) decreased (see Figure S13). The accuracy was also observed to be high with a good correlation to the results gained by LC-MS (*R*^2^ = 0.97), using the sliding recovery correction (eq 4) for the QFX results. There was no significant difference between the results from the LC-MS and the QFX (*p* = 0.63, using two-sided, paired *t*-test, *n* = 24), but the slope of the linear regression (QFX vs LC-MS) did indicate a slight general underestimation as with the 96-WP method (see Figure S14). With much fewer samples, the clinical sensitivity was still calculated to 80%, with 4 true positives and 1 false negative, while the specificity was calculated to 95%, with 18 true negatives and 1 false positive.

### Embryo Mortality versus Egg Size

As mentioned earlier, there seems to be a correlation, although weak, between the size of the eggs and the concentration of SumT (Figure S5A), and a clear separation can be seen between eggs weighing more than 120 mg or less than 120 mg (Figure S5B). In fact, the pathological limit of 0.19 nmol/egg, where a significant increase in mortality starts to occur, is close to the median concentration in eggs weighing less than 120 mg (0.23 nmol/egg) (Figure S5B). In fact, the lower quartile of eggs weighing less than 120 mg (0.08 nmol/egg) is below the LC_50_ (0.10 nmol/egg), indicating a significant mortality in this group. For eggs weighing more than 120 mg on the other hand, the lower quartile (0.28 nmol/egg) is above the highest concentration where a significant mortality increase is observed (0.19 nmol/egg), meaning that less than 25% of the samples from this group is at risk of increased mortality.

For salmon stocking purposes, this means that choosing only female specimens producing eggs larger than 120 mg for artificial breeding could produce good yields without the need for thiamine supplementation (bathing). However, the size of the eggs among the female salmon is not evenly distributed between different rivers, and some rivers have a large population of migrating females producing eggs smaller than 120 mg. Excluding these salmon from the breeding program would likely quickly result in a decrease in genetic diversity. In this case, the method presented here, with the QFX detector, could be used to quickly screen the eggs, hence enabling selection of eggs for breeding, based on thiamine content, thus reducing the need for thiamine supplementation without a large drop in yield. Ultimately, this may result in a stronger population, as the long-term effects of saving embryos that would die, by thiamine supplementation, are not well understood.

## Conclusions

The newly developed extraction procedure enables simple determination of thiamine in its different forms using LC-MS, together with the developed LC procedure, as well as large scale monitoring of the sum thiamine content in salmon roe using the newly developed 96-well plate method. The portable alternative to the 96 well plate method, using the DeNovix QFX, can easily be used within salmonid hatcheries or in field stations or research vessels. Extensive testing and evaluation of the methods ensure reliable use within the M74 monitoring program in Swedish rivers, and the results of the method validation were well within our set of requirements for the program.

## Supporting Information Available

Additional method details and results, including instrumental setting and additional data for the method validations (PDF)
