# Supplementary material for: Liquid Chromatography Mass Spectrometric Method and a Fluorometric 96 Well Plate Assay for Determination of Thiamine in Salmonid Eggs
Source: ACS Omega. 2024 Sep 27;9(40):41703–10. doi: 10.1021/acsomega.4c05862 (PMC11465547; doi:10.1021/acsomega.4c05862)
Supplement: Supplementary file 1 — ao4c05862_si_001.pdf [file ao4c05862_si_001.pdf]

Supporting Information

A liquid chromatography mass spectrometric method and a fluorometric 96 well plate assay for determination of thiamine in salmonid eggs

Manne Larsson[1,2], Lennart Balk[1], Elin Dahlgren[2], Efstathios Vryonidis[1], Dennis Lindqvist[1,*]

[1]Department of environmental science, Stockholm University, SE-106 91 Stockholm, Sweden

[2]Department of Aquatic Resources, Institute of Freshwater Research, Swedish University of Agriculture, SE-178 93, Drottningholm, Sweden

*Corresponding author: dennis.lindqvist@aces.su.se

13 Pages, including 15 Figures, and 6 Tables

LC-MS method

Instrumental settings

Table S1. Precursor and product ions for the different forms of thiamine and the internal standard, as well as their respective cone voltage and collision voltage.

Compund (quantifier/qualifier)	Precursor ion (*m/z*)	Product ion (*m/z*)	Cone (V)	Collision (V)
Thiamine (quant.)	264.90	121.85	30	15
Thiamine (qual.)	264.90	143.90	30	17
TMP (quant.)	344.70	121.85	30	15
TMP (qual.)	344.70	223.80	30	15
TDP (quant.)	424.80	121.85	71	22
TDP (qual.)	424.80	303.70	71	19
Amprolium (quant.)	242.95	149.90	30	14
Amprolium (qual.)	242.95	93.99	30	14

Fragmentation pathway

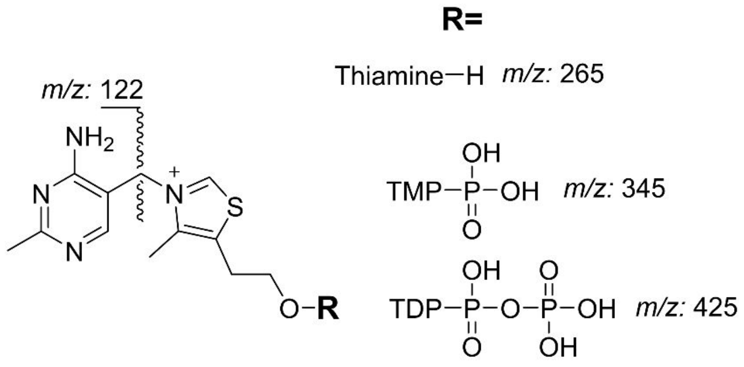

Figure S1. Major fragmentation pathway and m/z for thiamine and its analogues.

Accuracy

Table S2. Determined values of thiamine (nmol/g), in 20 roe samples, using the LC-MS method and corresponding values determined by a commercial laboratory.

Sample	New	Commercial	Average		
	Thiamine (nmol/g)		(nmol/g)	+25%	-25%
1	8.4	5.2	6.8	8.5	5.1
2	4.4	4.2	4.3	5.4	3.2
3	7.1	8.6	7.9	9.8	5.9
4	0.6	1.2	0.9	1.1	0.7
5	2.0	1.7	1.9	2.4	1.4
6	1.4	1.8	1.6	2.0	1.2
7	4.3	3.6	3.9	4.9	3.0
8	0.18	0.41	0.29	0.37	0.22
9	1.4	1.4	1.4	1.8	1.1
10	0.7	1.1	0.9	1.2	0.7
11	2.3	2.7	2.5	3.1	1.9
12	4.9	4.2	4.6	5.7	3.4
13	4.7	4.0	4.4	5.4	3.3
14	10.8	8.0	9.4	11.8	7.1
15	3.6	2.9	3.3	4.1	2.5
16	6.3	4.9	5.6	7.1	4.2
17	6.4	6.2	6.3	7.9	4.7
18	3.7	4.4	4.1	5.1	3.1
19	2.9	3.2	3.1	3.8	2.3
20	3.6	4.4	4.0	5.0	3.0
2-sided	paired	*t*-test			
p =	0.29	>0.05			

Grey values were outside of ± 25% of the average value

Precision

Table S3. Precision was determined by conduction eight analyses of three different samples and determining average values (Av), standard deviation (STDev) and coefficient of variance (CV%).

	**Sample 1**			**Sample 2**			**Sample 3**		
	Thia.	TMP	TDP	Thia.	TMP	TDP	Thia.	TMP	TDP
	(nmol/g)			(nmol/g)			(nmol/g)		
1	1.65	0.16	0.27	5.43	0.13	0.32	4.56	0.15	0.45
2	1.53	0.16	0.28	4.44	0.13	0.31	5.16	0.14	0.47
3	1.62	0.15	0.26	5.72	0.13	0.26	4.82	0.12	0.50
4	1.48	0.16	0.24	5.51	0.13	0.29	4.40	0.12	0.52
5	1.81	0.15	0.26	5.45	0.11	0.34	4.48	0.15	0.50
6	1.72	0.15	0.30	5.55	0.11	0.33	4.87	0.13	0.34
7	1.99	0.16	0.29	5.58	0.11	0.35	4.94	0.13	0.54
8	1.79	0.17	0.26	5.99	0.13	0.31	4.27	0.12	0.29
**Av**	1.70	0.16	0.27	5.46	0.12	0.31	4.69	0.13	0.45
**STDev**	0.17	0.01	0.02	0.45	0.01	0.03	0.31	0.01	0.09
**CV%**	9.8	4.3	7.5	8.2	7.7	8.9	6.5	9.5	20.0

Linearity

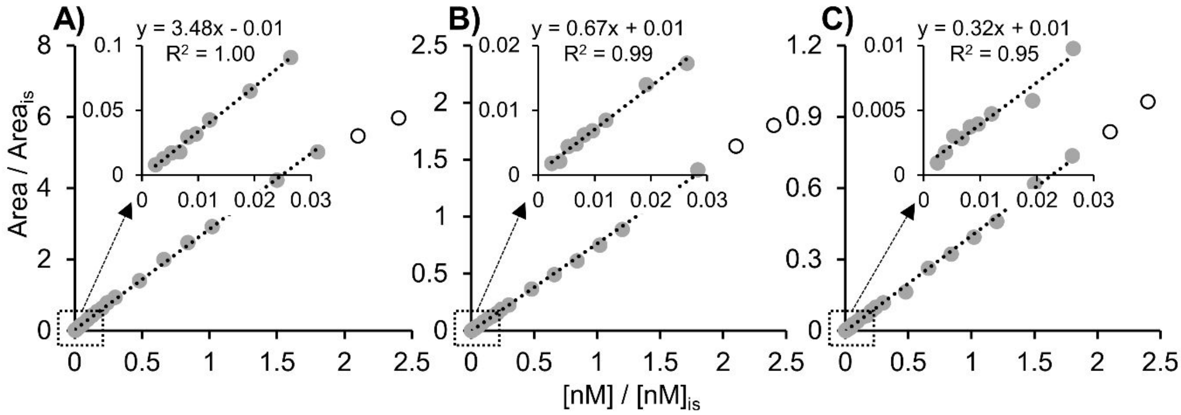

Figure S2. Calibration curve displaying the dynamic range on the LC-MS system, the lower part of the curves used for determining LOD and LOQ are magnified and inserted top left. **A)** Thiamine. **B)** TMP. **C)** TDP.

Fluorometric 96 well plate assay

8-channel plunger

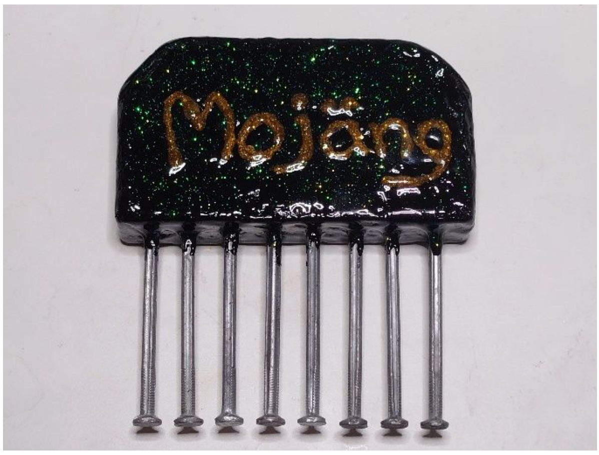

Figure S3. Picture of the 8-channel plunger, consisting of 8 zinc plated nails inserted in a wooden handle covered in epoxy.

Fluorescence spectra

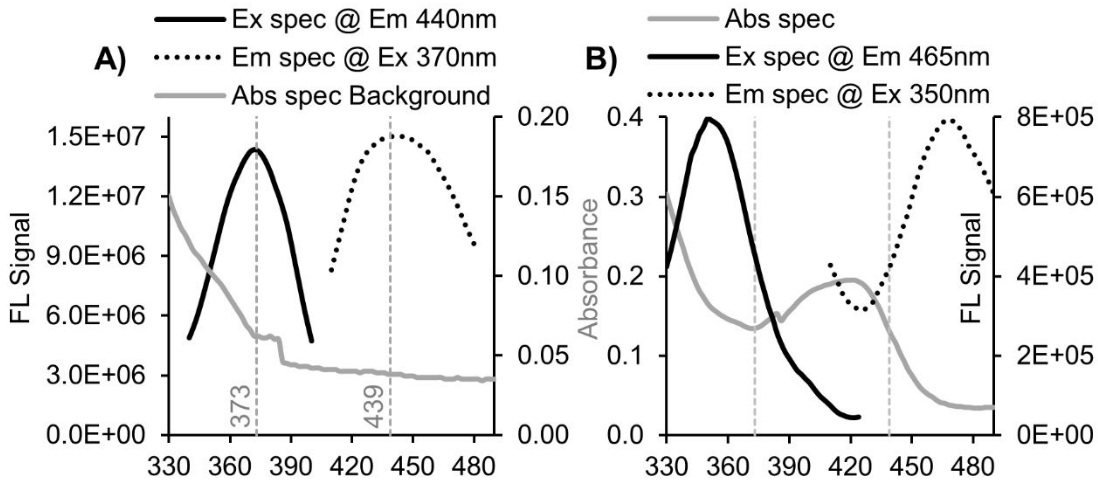

Figure S4. A) Excitation and emission spectra for TiOC as well as absorbance spectra of the background. **B)** Excitation, emission, and absorbance spectra of concentrated oxidation reagent $K_3[Fe(CN)_6]$ in the absence of thiochrome.

Thiamine levels and size of the eggs

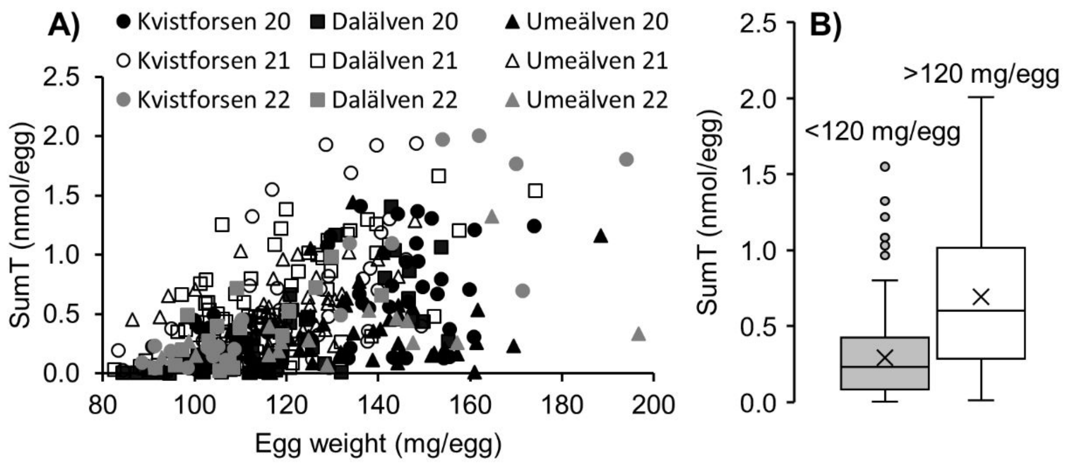

Figure S5. A) SumT versus weight of the eggs. **B)** Box plot displaying SumT in samples weighing more or less than 120 mg. Based on samples from 3 rivers and 3 different years, total 382 samples.

Recoveries

Table S4. A) Recovery (%) of TMP and TDP relative that of Thiamine from spiked procedural blanks. **B)** Recovery (%) of spiked TiOC in the IPR partition step, each concentration was tested 3 times.

**A)**	**Relative Rec. (%)**		**B)**	**TiOC**	**Rec.**		**STDev**
	TMP	TDP		(nmol)	(%)		
	94	60		0.2	84	±	3.1
	88	64		0.4	94	±	0.6
	104	70		0.8	95	±	1.3
	86	55		1.1	92	±	0.4
	90	59		1.5	94	±	1.0
	94	68		2.3	93	±	1.5
	91	61		3.0	91	±	0.1
	97	70		3.8	88	±	2.5
	93 ± 5.6	63 ± 5.3		Av.	91		

Stability of extracts

The stability of the oxidized extracts in IPR were evaluated by comparing the fluorescent signal, over time (day: 0, 5, 14, and 28), from six extracted samples, each, stored in ambient temperature (20°C), refrigerator (8°C), and freezer (-20°C).

No statistically significant differences over time were observed in extracts stored in a freezer at -20°C (p = 0.33 day 5; 0.64 day 14; 0.59 day 28) or a refrigerator at 8°C (p = 0.19 day 5; 0.21 day 14; 0.51 day 28) using paired two-tailed t-tests of the FL signal relative that registered at day 0. When stored at room temperature a small increase in signal was registered after 14 days, this then decreased again up to day 28 but was still significant (p = 0.08 day 5; 0.007 day 14; 0.007 day 28), see table S5.

Table S5. FL signal of TiOC over time relative to that registered at day 0, set to 100%. 6 samples stored at -20°C, 8°C, and 20°C respectively. All samples were stored in the dark.

	**No.**	**Day 0**	**Day 5**	**Day 14**	**Day 28**
Freezer -20°C	1	100	95	107	111
	2	100	110	114	97
	3	100	98	110	106
	4	100	100	109	106
	5	100	96	103	102
	6	100	89	90	94
Refrigerator 8°C	1	100	115	126	100
	2	100	115	123	99
	3	100	112	116	92
	4	100	109	111	88
	5	100	97	98	94
	6	100	97	95	87
Room temp. 20°C	1	100	116	126	118
	2	100	116	120	103
	3	100	119	130	109
	4	100	114	121	104
	5	100	91	100	108
	6	100	109	115	103

Thiamine versus TMP and TDP in S. salar eggs

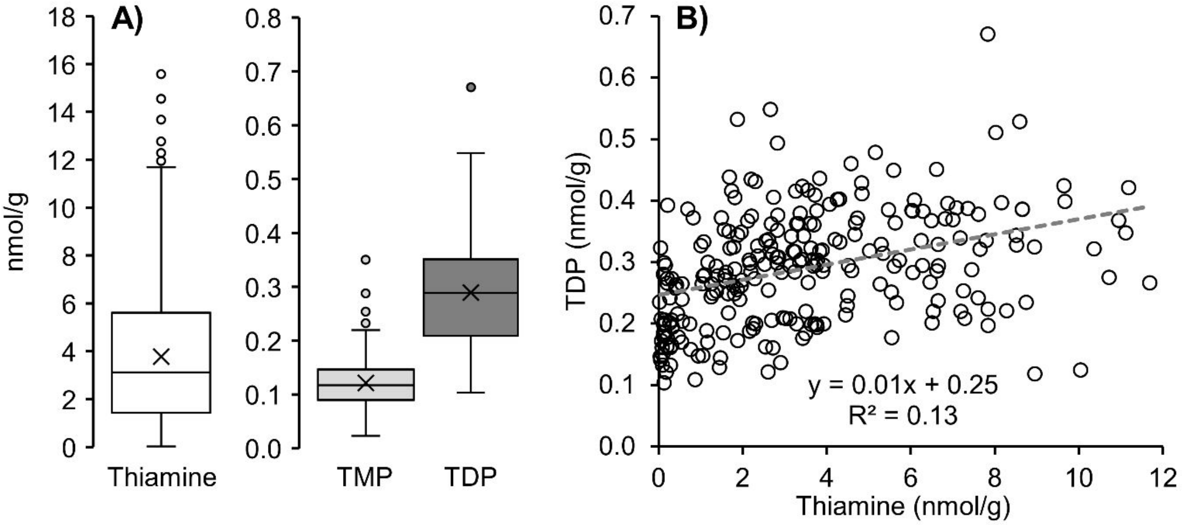

Figure S6. A) Levels of thiamine, TMP, and TDP in *S. salar* roe based on 2 years (2021-2022) and 3 rivers, total 257 samples. **B)** Correlation between the levels of thiamine and TDP in salmon roe, displaying fairly constant levels of TDP in relation to thiamine, a similar correlation is seen between TMP and thiamine with y = 0.01x + 0.09 and $R^2 = 0.32$.

Optimization of oxidation reagent amount

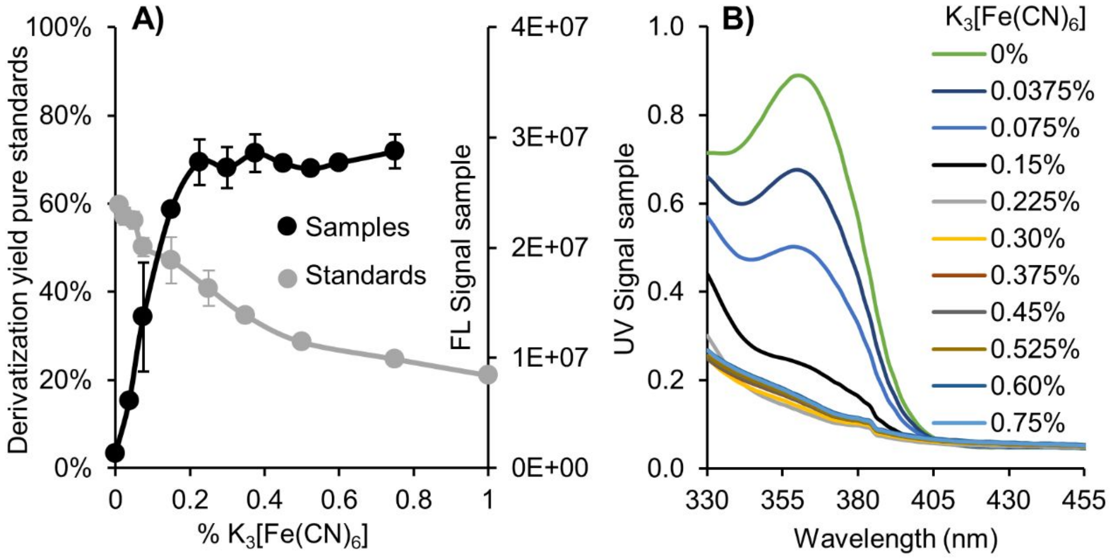

Figure S7. A) Oxidation yields dependence on the amount of reagent, evaluated using pure standards (grey, left y-axis). Gained signal from repeated analyses of eggs from the same female using different amount of oxidation reagent (black, right y-axis). **B)** UV background spectra from samples from the same female using different amounts of oxidation reagent. Based on 2-3 analyses per reagent concentration. Amount of reagent is the concentration (%) of $K_3[Fe(CN_6)]$ in the 50 μL KOH (0.5 M) solution added to the sample (200 μL), and not final concentration.

Precision

Table S6. Precision evaluation through 8 quantitative analyses of eggs from the same female. Determined average levels (nmol/egg), standard deviation of the 8 analyses, and coefficient of variance. In total 8 females were used, eggs from one female were analyzed using both the 96-deep well plates and μ-tubes.

	n	Av	STDev	CV%
96-WP	8	0.15	0.03	23
96-WP	8	0.17	0.03	15
96-WP	8	0.30	0.05	18
96-WP	8	0.45	0.09	19
96-WP	8	0.51	0.10	19
96-WP	8	0.54	0.09	16
96-WP	8	0.61	0.07	12
96-WP	8	1.26	0.14	11
μ-Tube	8	0.50	0.04	7.8

Grey samples belonged to the same female.

Linearity

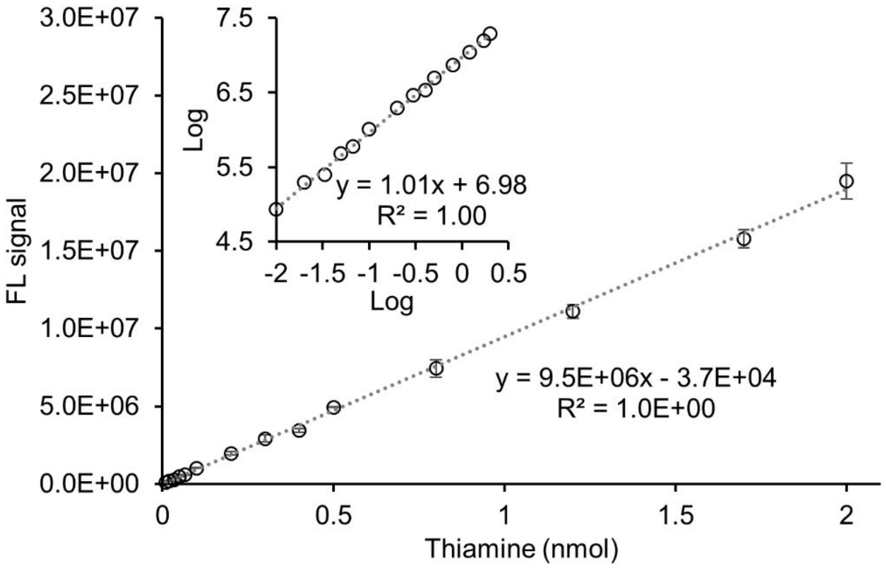

Figure S8. Calibration curve for the 96-WP method, analyzed on the iD3 plate reader (Molecular Devices). The amounts on the x-axis represent the amount of thiamine added to each well prior to extraction and oxidation, i.e., not final concentration. Results based on triplicate extractions. The inserted graph displays the calibration curve in logarithmic scale for further visualization of the linearity.

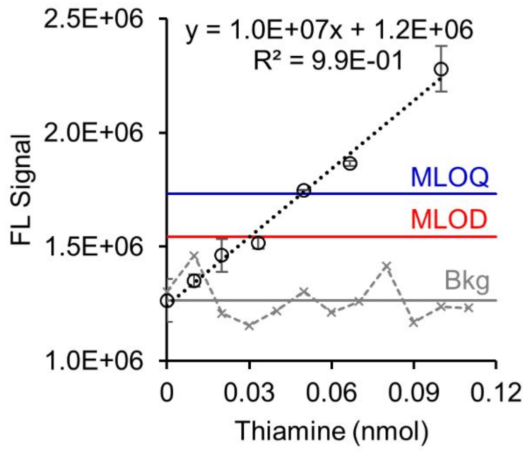

Figure S9. The lower section of the calibration curve in Figure S8. Grey solid line displays the average background signal, with the dashed line with markers displaying the individual background analyses (these have no connection to the x-axis but is rather just spread out for visualization). Red line depicts the MLOD (3× the standard deviation of the background added to the average background). Blue line depicts MLOQ (5× the standard deviation of the background added to the average background.

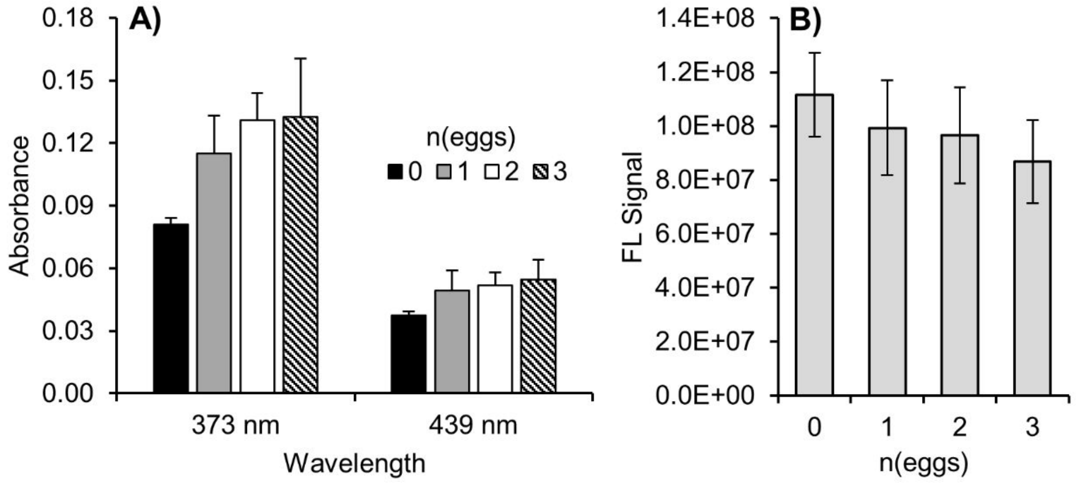

Quenching and background

Figure S10. A) Background absorbance (at 373 and 439 nm) caused by different number of eggs with low levels of native SumT, extracted and oxidized according to the presented method. **B**) Quenching of the FL signal caused by the absorbance of the matrix, using different number or eggs to sequentially increase the background. The eggs contained low native levels of SumT and the final 200 μL extract were spiked with TiOC (10 μM, 40 μL) to determine the quenching effect on the signal by the egg extract. 4 analyses were conducted per set. The background absorbance from 2 eggs were statistically significantly higher than with 0 eggs ($p = 0.0001$, λ 373 nm; $p = 0.002$, λ 439 nm), but it did not

significantly differ from the background from 1 egg ($p = 0.10$, λ 373 nm; $p = 0.34$, λ 439 nm). The FL signal from the spiked 2 egg extracts did not significantly differ from that given by 0 eggs ($p = 0.12$) indicating little background quenching from the extract. One-sided *t*-tests were used to evaluate differences.

Embryo mortality and basis for expressing levels

S. salar egg thiamine levels and embryo mortality

The LC_{50} for embryo mortality, based on thiamine levels in eggs prior to fertilization, was calculated with GraphPad Prism 8.3.0, using a robust regression, four parameter variable slope, Log[SumT] vs. Mortality. Data was collected from Werner et al. (2006), SumT (nmol/g ww) pre-fertilized eggs.

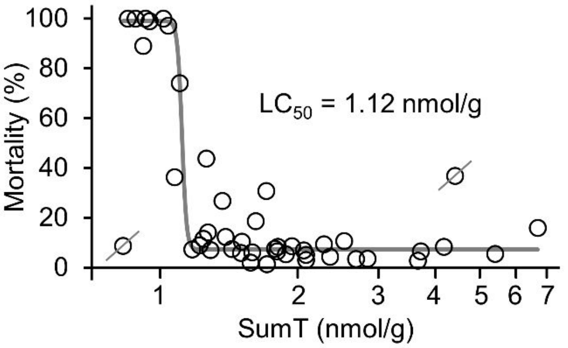

Figure S11. Dose-response curve depicting embryo mortality dependence on SumT concentration in pre-fertilized eggs. Data from Werner et al. (2006).

Egg thiamine levels, nmol/g ww versus nmol/egg

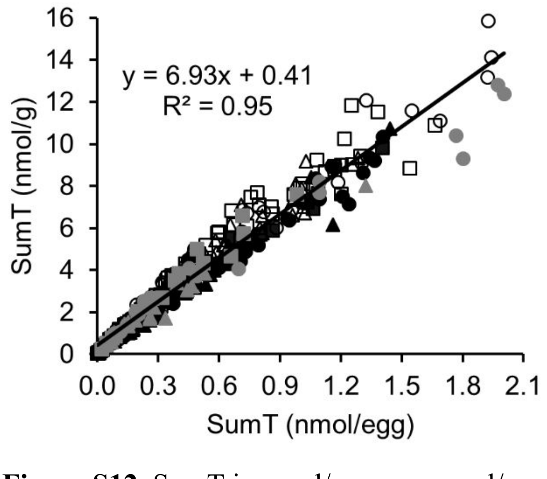

Figure S12. SumT in nmol/g versus nmol/egg. Based on samples from 3 rivers and 3 different years, a total of 382 samples.

Portable fluorometric detection option

QFX linearity and sensitivity

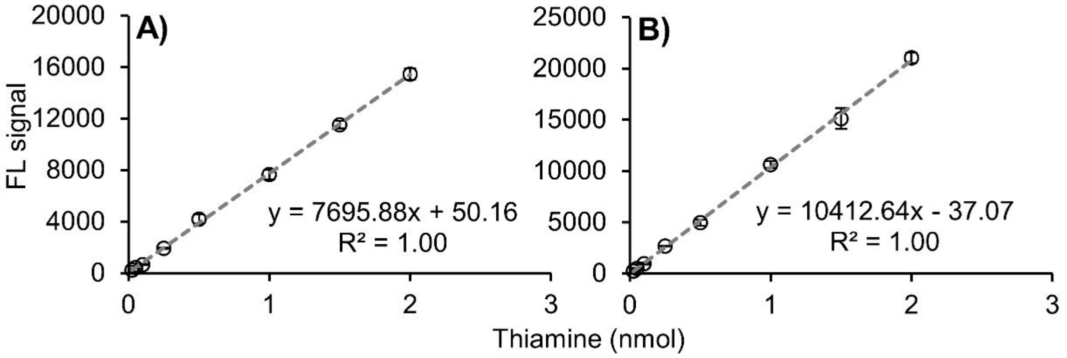

Figure S13. Nine-point calibration curves (including 0), each datapoint is based on 4 analyzes (spiked procedural blanks), except 0, which is based on 14. Amounts on the x-axis represent the amount of thiamine added to each blank. **A)** Analyzed using 0.5 mL graded μ-tubes (Eppendorf), MLOD and MLOQ were calculated to 0.008 and 0.015 nmol/egg respectively. **B)** Analyzed using Axygen 0.5 mL thin wall, clear PCR tubes (Corning Inc.), MLOD and MLOQ were calculated to 0.006 and 0.009 nmol/egg respectively.

QFX accuracy

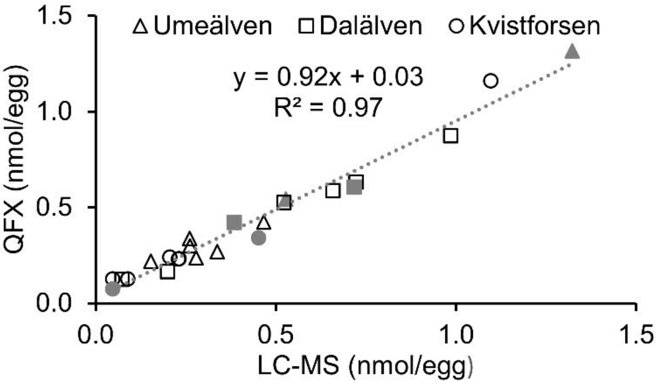

Figure S14. Quantitative determination of SumT using μ-tube extraction and fluorescence measurement using the DeNovix QFX versus determination using the LC-MS based method. Grey marks were analyzed in the QFX using the Eppendorf 0.5 mL μ-tubes, while the rest were analyzed using the clear, thin wall PCR tubes.

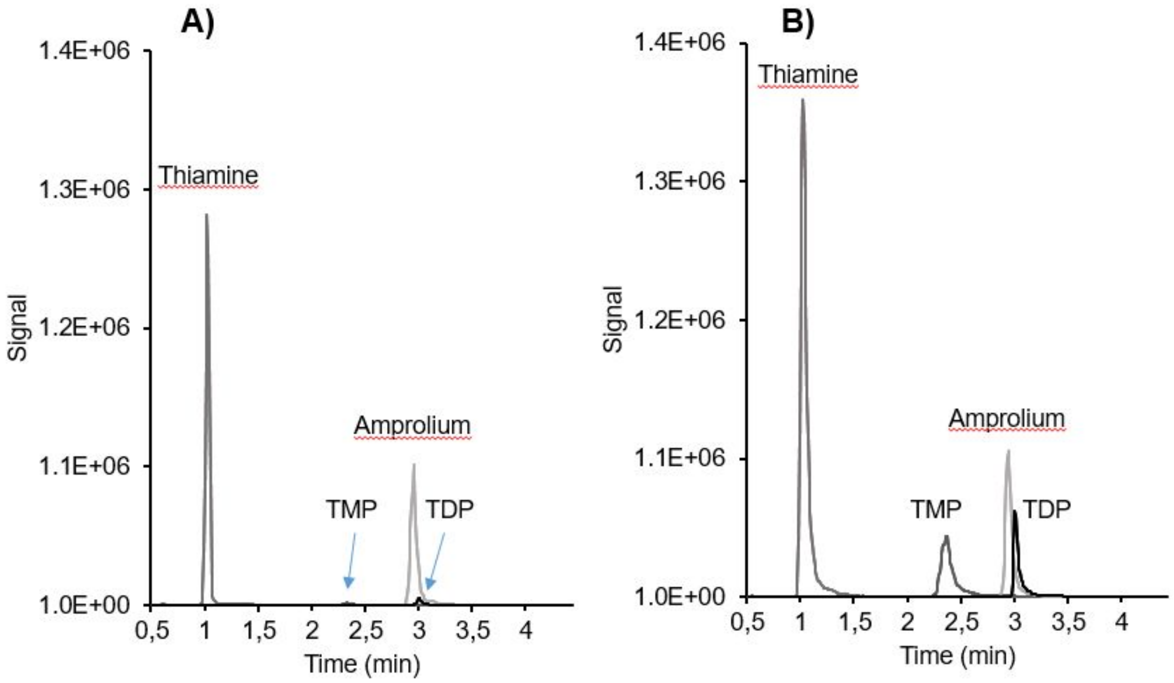

Figure S15. Example chromatogram from the LC-MS method. **A)** Representative chromatogram of an actual sample. **B)** Pure standards at a concentration of 1.00 µM, for reference.

References

Werner M.R., Rook B., Greil R. Egg-thiamine Status and Occurrence of Early Mortality Syndrome (EMS) in Atlantic Salmon from the St. Marys River, Michigan. *J. Great Lakes Res.* 32:293–305. Internat. Assoc. Great Lakes Res., 2006.
